# A, B, and C Rhinoviruses: New Knowledge from an Impressive Consortium. A Step Forward for Rhinovirus Vaccine Efforts or a Step Back?

**DOI:** 10.1164/rccm.202102-0346ED

**Published:** 2021-04-01

**Authors:** Sebastian L. Johnston

**Affiliations:** ^1^ National Heart and Lung Institute Imperial College London London, United Kingdom and; ^2^ Asthma UK Centre in Allergic Mechanisms of Asthma London, United Kingdom

Rhinovirus (RV) infections cause asymptomatic infections, wheezing, and nonwheezing lower respiratory tract infections in young children (1) and the majority of acute attacks of asthma (2) and chronic obstructive pulmonary disease (3), resulting in substantial morbidity and deaths. There are 157 numbered RVs, which are divided into A, B, and C species based on sequence homology (4). One hundred RV-As and RV-Bs have been serotyped, and most do not crossneutralize (5). Studies with RV-Cs have been prevented by difficulties growing these viruses, but given the sequence divergence between A/B and C strains, it is unlikely the 51 numbered C strains will crossneutralize. The need for >150 strains in RV vaccines has hampered vaccine development. However, if certain RVs were more common causes of severe disease, could vaccine efforts be focused on these RVs to help move vaccine development forward?

RV-Bs are less likely to cause severe illness in children than RV-A or RV-C (1). However, data on RV-A/Cs and severe respiratory illnesses are not consistent, as studies in children have reported more RV-Cs than RV-As (6), whereas studies in adults reported more RV-As than RV-Cs (7).

In this issue of the *Journal*, Choi and colleagues (pp. 822–830) have made significant progress in understanding the importance of different RV species and strains in respiratory illnesses in children (8). They analyzed nasal and plasma samples from birth to age 18 in the COAST (Childhood Origins of ASThma) study, which studied 289 children from Madison, Wisconsin, at birth, 210 of whom were followed to age 18. They partially sequenced >8,000 RV-positive samples from asymptomatic and illness visits to compare RV-A and RV-C frequencies at ages 0–3, 4–8, 9–13, and 14–18 and found that RV-A and RV-C were similarly common at ages 0–3, but thereafter, RV-A was approximately twice as common as RV-C (*P* < 0.001). The authors hypothesized that this change in frequency might result from differences in neutralizing antibody (nAb) responses with age, as nAbs protect against reinfection with RVs. nAbs to RV-C had not previously been analyzed because infection of permissive cells with RV-Cs *in vitro* does not result in visible cytopathic effect (CPE), so there was no suitable system to assay infectivity and its neutralization by observing CPE.

The authors therefore developed a novel RV-C neutralization assay using RV-C2, RV-C15, and RV-C41, which were clinical isolates they had cloned and produced as live viruses by reverse genetics. These RV-Cs were preincubated with serial dilutions of plasma and inoculated onto HeLa-E8 cells, which are permissive to RV-C replication (9). Viral replication was measured by qPCR and inhibitory concentration 50% (IC_50_) nAb titers calculated.

The same qPCR-based assay was used to measure IC_50_ nAb titers to RV-A16, which were then compared with standard neutralization titers against RV-A16, measured by CPE visualization using traditional methods to generate tissue culture infective dose 50% (TCID_50_) titers. IC_50_ and TCID_50_ nAb titers correlated very well (*r*_s_ = 0.83, *P* = 0.006), thus validating this novel and very useful tool for studying RV-Cs.

The authors then analyzed nAbs to RV-A7, RV-A16, RV-A36, and the three RV-Cs in plasma from 20 COAST study participants, measured at 2, 10, and 16 years. At age 2, only 5% of samples had nAbs to any of the three RV-As, whereas 27% had nAbs to the three RV-Cs. The corresponding figures for age 10 were 25% and 70%, and for age 16, they were 18% and 78% (*P* < 0.001 at each age). Thus, nAbs to these RV-Cs were much more common and much more durable to age 16 than nAbs to the three RV-As.

The low frequencies of nAbs against the RV-As may have been skewed by very low frequencies of titers against RV-A7 at all ages tested, as these were present in only 5% at ages 2 and 10 and 10% at age 16, suggesting this strain was possibly unrepresentative because it was clearly much less prevalent in the Wisconsin area during recruitment to COAST than RV-A16 and RV-A36, which each had titer frequencies of 35% at age 10. The frequency of 35% at age 10 for RV-A16 is consistent with the experience that ∼50% of adults have detectable nAbs against RV-A16.

The low frequencies of nAbs against the RV-As may also have been skewed by unexpectedly low frequencies of titers against RV-A16 at age 16, as these were only 10%, considerably lower than at age 10 and against RV-A36 at age 16 (both 35%). Thus, more representative frequencies for RV-A strains would likely be ∼35% at age 10 and ∼35% or higher at age 16. Nonetheless, such figures are still considerably lower than those against the RV-Cs at the same ages (70% and 78%). The number of RVs tested was low (3 RV-As and 3 RV-Cs) and the number of samples was low (20 at each age). More data will be needed to confirm these findings and to extend them into greater numbers of RVs and children and into adulthood, but the authors’ conclusions that RV-Cs become less common with age because of development of higher titers of durable nAbs is interesting and provides a logical explanation for the prevalence data in childhood and adulthood (6, 7).

A further interesting finding was the detection of 94% of known RV-As in the COAST study analysis, indicating there has been very little change in circulating RV-A strains over ∼50–60 years (the RV-As were characterized and numbered in the late 1960s to early 1980s) (5). They also detected 98% of known RV-Cs, but this is less surprising, as the RV-Cs were characterized in the last 10–15 years and to a large degree in samples from the COAST study (10). Further analyses of RV-Cs in future years and in different study populations will be needed to inform on turnover of RV-C strains over time.

The authors then collected 17,664 samples from 14 cohorts studying children from birth to 18 years in the United States, Finland, and Australia; 10,185 samples were positive for RV, 6,643 from sick visits and 3,542 from asymptomatic visits, allowing for the investigation of frequencies in sickness and in health. There were slightly more positives during sick visits for RV-Cs (72.4%) than RV-As (66%), whereas only 37.8% of RV-Bs were at sick visits. Thus, at least in children, RV-Bs were strikingly less pathogenic than either A or C, and RV-Bs were detected more frequently on asymptomatic than on symptomatic visits.

The authors detected 178 RV types, identifying 21 more RV strains circulating than previously identified (157) (4). They also showed certain strains (RV-A12, 78, and 101 and RV-C02 and 11) were more commonly detected consistently over the 12-year time span of sample collection. However, the most frequently detected type (RV-A78) was only detected in 2.7% of samples, so no single RV strain accounted for more than a very small percentage of RV infections.

Consistent with the COAST findings, RV-Cs were more common than RV-As until 4–5 years old, but thereafter, RV-A infections became progressively more common, outnumbering RV-C by ∼2:1 from age ∼15.

Consistent with previous reports, RV-Cs were more likely associated with wheeze, and the CDHR3 rs6967330 asthma risk allele significantly increased the risk of RV-C illness, both relationships being independent of age.

I congratulate the authors for performing these extensive and detailed investigations and for significantly extending our understanding of RV species in childhood. I hope their work will stimulate further similar work in different age groups and with greater numbers of virus strains to increase our understanding even further.

The authors hope their work may assist vaccine development strategies for RV-C strains for use in early life. However, by *1*) showing that RV-A strains outnumber RV-C strains by 2:1 after age 15, and thus that RV-A vaccines are desperately needed in addition to RV-C vaccines, and *2*) identifying that 21 more RV strains than previously known were actively circulating (as were almost all the previously known strains), thus requiring any pan-RV vaccine to cover 178 strains rather than 157 strains, they may have made the job of actually developing effective RV vaccines that little bit harder!

## Supplementary Material

Supplements

Author disclosures
