# Supplementary material for: A, B, and C Rhinoviruses: New Knowledge from an Impressive Consortium. A Step Forward for Rhinovirus Vaccine Efforts or a Step Back?
Source: Am J Respir Crit Care Med. 2021 Apr 1;203(7):786–8. doi: 10.1164/rccm.202102-0346ED (PMC8017583; doi:10.1164/rccm.202102-0346ED)
Supplement: Supplements [file rccm.202102-0346ED.html]

A, B, and C Rhinoviruses: New Knowledge from an Impressive Consortium. A Step Forward for Rhinovirus Vaccine Efforts or a Step Back? | American Journal of Respiratory and Critical Care Medicine

- disclosures.pdf (150 KB)
